# ADHD-associated risk taking is linked to exaggerated views of the benefits of positive outcomes

**DOI:** 10.1038/srep34833

**Published:** 2016-10-11

**Authors:** Rachel Shoham, Edmund J. S. Sonuga-Barke, Hamutal Aloni, Ilan Yaniv, Yehuda Pollak

**Affiliations:** 1 Department of Psychology, The Hebrew University of Jerusalem, Jerusalem, Israel; 2 Special Education department, Talpiot College, Holon, Israel; 3 Developmental Brain-Behaviour Laboratory, University of Southampton, UK; 4 Department of Experimental, Clinical and Health Psychology, Ghent University, Belgium; 5 Federmann Center for Study of Rationality, The Hebrew University of Jerusalem, Jerusalem, Israel; 6 The Seymour Fox School of Education, The Hebrew University of Jerusalem, Jerusalem, Israel

## Abstract

Attention deficit and hyperactivity disorder (ADHD) is often assumed to be associated with increased engagement in risk-taking behaviors. The current study sought to understand the mental processes underlying this association using a theory-driven behavioral economics perspective. Psychological risk-return models suggest that risk and benefit are inherently subjective, and risk taking is best understood as the interplay between cognitions and motivations regarding the benefits and risks of alternatives. A sample of 244 adults was assessed for ADHD symptoms. The likelihood of engagement in a range of risky behaviors (e.g., driving without wearing a seat belt), the magnitude of perceived benefit and risk ascribed to these behaviors, and benefit and risk attitudes of each participant were extracted from the Domain Specific Risk Taking (DOSPERT) scales. ADHD symptoms were correlated with more risky behaviors and perception of greater benefits from engaging in these behaviors, but were not correlated with risk perception. Mediation analysis revealed that the association between ADHD symptoms and engagement in risk taking was mediated by perceived benefits. These findings highlight the idea that people with high level ADHD symptoms tend to engage in risky behaviors because they find such behavior particularly appealing, rather than because they seek risk per se.

Attention deficit and hyperactivity disorder (ADHD) is a neurodevelopmental disorder, characterized by a persistent pattern of inattentive, hyperactive and impulsive behavior, interfering with educational, social and occupational functioning^1^,^2^. Individuals diagnosed with ADHD tend to engage deliberately in behaviors that place them at risk for negative outcomes^3^ including smoking^4^, substance abuse^5^,^6^,^7^, dangerous driving^8^, gambling^9^ and unprotected sex^10^.

The present study takes the perspective of a behavioral economics^11^,^12^ in an attempt to understand the mental processes that might account for risk taking in ADHD. Behavioral economic approaches see the individual as an active agent who makes preferences based on calculation of the expected utility of the available alternatives. According to the normative expected utility framework, the expected value of a risky alternative comprises its subjective potential payoff weighted by its probability. A rational decision maker should calculate the expected value of each available alternative and consistently choose the one with the highest expected value. An important construct of this theory is *Risk attitude*, namely, a person’s position on the continuum from *risk aversion* to *risk seeking*; risk attitude is reflected in the shape of the person’s utility function, which is commonly considered a personality trait^13^.

In proposing their version of behavioral decision theory (BDT), Weber, Blais, and Betz’s^11^ maintained that risk and benefit are inherently subjective, and that individuals make decisions based on their perceptions of the costs and benefits of the alternatives as well as their attitudes toward these perceived cost and benefit^11^. Risk/benefit perception is defined as the magnitude of riskiness/benefit a person ascribes to an alternative, whereas perceived risk/benefit attitude refers to how much the person is attracted or repelled by her perceived risk/benefit, or how much her risk taking is affected by her risk and benefit perceptions^11^. People may take risks because they perceive the risk to be low and/or the benefits to be high, or alternatively, because their risk aversion is low and/or their benefit seeking is high. Perceived benefit often elicits attraction, whereas perceived risk evokes repulsion. However, the extent of attraction and repulsion differs across people; sometimes perceived risk may be intrinsically attractive (e.g., sensation seeking). These approaches are captured in Weber *et al*.’s BDT in the following regression equation:


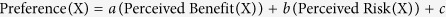


In this equation, preference for alternative X is a function of the tradeoff between the perceptions of benefit and risk associated with that alternative as well as the person’s general attitudes toward benefit and risk (coefficients *a* and *b*, respectively). Measures of perceptions of benefit and risk are based on self-reports, whereas the attitudes towards benefit and risk are calculated by regressing risk taking behavior on risk perception and benefit perception separately for each individual, and calculating the respective coefficients. The distinction between perception and attitude is crucial for understanding human decision-making according to Weber *et al*. Studies have indeed shown systematic individual, group, and cultural differences in perceptions of risks and benefits^14^,^15^,^16^. Attitudes towards perceived risk are less affected by context, but they still vary across domains^17^.

To date the role of risk and benefit perceptions of risk-taking behavior in ADHD has not been studied from this BDT perspective, which distinguishes between perceptions and attitudes. More generally, studies so far suggest that individuals with ADHD do not seek risks per se^18^, but they perceive the outcomes of risk taking behaviors to be either particularly appealing or less risky. In one study, children with ADHD attributed less severe consequences to risky activities^19^. A qualitative study showed that children with ADHD overestimated their physical abilities and disregarded negative consequences of their risk taking^20^. Among adolescents, higher inattention rates were correlated with less negative expectancies regarding the outcomes of cigarette smoking, whereas higher hyperactivity/impulsivity rates were correlated with positive expectancies about smoking^21^. Pedersen, Harty, Pelham, Gnagy, and Molina^22^ found that children with ADHD had lower levels of negative alcohol expectancies, though they also had lower levels of positive expectancies, compared with controls^22^. The finding of different perceptions of the outcomes is consistent with the idea that individuals with ADHD have a “positive illusory bias”, i.e. the tendency to provide overly positive reports of their own competence^23^.

In this paper, we report a study using the Domain Specific Risk Taking (DOSPERT) scale. The scale was developed by Blais and Weber^24^ to assess risk taking (an aggregate measure based on likelihood estimates of engaging in 30 different risky behaviors), benefit perception (an aggregate measure based on perception of the benefits received from engaging in each of the 30 behaviors) and risk perception (the level of risk associated with each of the 30 behaviors)^24^. Each of the three DOSPERT scales is divided into five different domains of risk-taking (health and safety, financial, recreational, social, and ethical). For our primary analyses in this research, we adopted a dimensional conceptualization of ADHD, consistent with current taxometric^25^ and genetic^26^ evidence. We hypothesized that (1) ADHD symptoms would be associated with risk taking in all domains, and that perception, rather than attitude, would (2) correlate with ADHD symptoms, and (3) mediate ADHD-related risk taking.

## Results

### Preliminary analysis

Table 1 presents descriptive statistics for the ASRS and DOSPERT scores. Normality testing using Kolmogorov-Smirnov test revealed that the following variables, age, ASRS total and sub-dimensions’ scores, the DOSPERT calculated total score of perceived benefit attitude, as well as most of the DOSPERT specific domain scores were not distributed normally. For the sake of simplicity, we used the non-parametric statistics to describe and analyze the correlations among all demographic variables, ASRS scales (general, inattention and hyperactivity/impulsivity) and DOSPERT scores. Male and younger participants reported higher risk taking and benefit perception, compared to female and older participants. Consequently, gender and age were used as covariates in mediation analyses. Years of education did not correlate with the DOSPERT scales and was not included in further analyses.

### Correlational analysis

Non-parametric correlations among the primary study variables are presented in Table 2. As expected, among the DOSPERT scales, level of benefit perception positively correlated with level of risk taking, and level of risk perception negatively correlated with level of risk taking. In addition, benefit perception negatively correlated with risk perception. ASRS scores positively correlated with levels of risk taking and benefit perception, and negatively correlated with levels of perceived-benefit attitude. In contrast, ASRS score did not correlate with levels of risk perception and perceived-risk attitude.

Inattention and hyperactivity scores showed similar pattern of correlations with the DOSPERT scales, namely, positive correlation with risk taking and benefit perception, negative correlation with perceived-benefit attitude, and no correlation with risk perception and perceived-risk attitude (see Table 2).

### Mediation analysis

The primary analysis examined whether the DOSPERT total scores of benefit and risk perception, as well as the derived perceived-benefit attitude and perceived-risk attitude, mediated the relation between the ASRS total score and the DOSPERT total score of risk taking, including age and gender as covariates.

**Figure 1:**
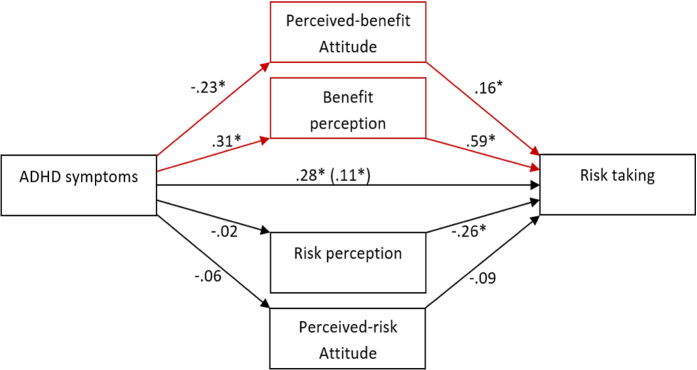
Final mediation path analysis predicting risky behavior. Values reflect standardized regression coefficients of direct and indirect effects of ADHD on risky behavior. The unstandardized regression coefficient of the direct effect after considering other mediators is presented in parentheses. The covariates of age and gender are not shown in the figure for visual clarity. *N* = 244 (132 females, 112 males). *p < 0.05.

The path analysis in Fig. 1 depicts the direct effects and indirect pathways for the contribution of ADHD symptoms on risk taking through its effects on benefit and risk perception. Together the model accounted for 57.3% of the variability in risk behavior (*P* < 0.0001). The standardized regression coefficients between ADHD symptoms and risk taking before considering mediators, between ADHD symptoms and benefit perception, and between ADHD symptoms and perceived-benefit attitude were statistically significant (*P* < 0.001). The bootstrapped standardized indirect effect mediated by benefit perception and by perceived-benefit attitude were significant. The indirect effect of ADHD symptoms, mediated by risk perception and perceived-risk attitude were not significant. ADHD symptoms still predicted risk taking after accounting for the indirect effect (see Table 3 for coefficients and CIs).

Additionally, similar mediation analysis was conducted separately for ASRS sub-dimensions. Mediation analysis for each ASRS dimension revealed significant indirect effects mediated by benefit perception and by perceived-benefit attitude. The indirect effect of ADHD symptoms, mediated by risk perception and perceived-risk attitude were not significant. The direct effect of the inattention scores on risk taking was marginally significant, whereas the direct effect of hyperactivity was significant (see Table 3 for coefficients and CIs).

### Risk taking domains

Finally, similar mediation analyses were repeated for each domain separately (see Table 4). As noted, perceived risk/benefit attitude were not calculated for each domain and were not included in the model. For four domains, i.e., health/safety, recreational, financial and ethical, ASRS scores positively correlated with levels of risk taking and benefit perception, but not with levels of risk perception. Separate mediation analyses for these four domain revealed indirect effects in which benefit perception, but not risk perception, mediated the association between ASRS score and risk taking. The direct effect of the ASRS score on risk taking remained significant for the health/safety and the ethical domains. On the other hand, regarding the social domain, ASRS scores positively correlated with levels of risk perception, but not with levels of risk taking and benefit perception. Separate mediation analyses for the social domain revealed indirect effects in which risk perception, but not benefit perception, mediated the association between ASRS score and risk taking. The direct effect of the ASRS score on risk taking was not significant (see Table 4 for coefficients and CIs).

## Discussion

This is the first study to examine associations between ADHD symptoms, engagement in real-life risky behaviors and perceptions regarding the benefit and risk of these behaviors. The following discussion will focus on two general findings: 1. ADHD symptoms predicted self-reported engagement in various domains of risk taking behavior. 2. Higher benefit perception of risk taking behaviors, but lower perceived-benefit attitude, correlated with ADHD symptoms and mediated engagement in risk taking behavior.

### ADHD symptoms and risk taking behavior

Self-reported ADHD symptoms of inattention and hyperactivity/impulsivity predicted self-reported engagement in a variety of risky behaviors. These findings are in agreement with many studies documenting increased risk taking by people with ADHD. However, most of the studies compared groups of participants with and without clinically diagnosed ADHD. Only seldom do researchers investigate the relation between ADHD symptoms and risk taking in the general population. For example, Kollins *et al*.^4^ found that each reported inattention and hyperactivity/impulsivity symptom significantly increased the likelihood of regular smoking^4^, and Pingault *et al*.^27^ reported on a prospective population cohort that inattention predicted nicotine dependence^27^. Our study extends studies such as these on ADHD-related cigarette smoking into risky behavior in general.

Most of the studies reporting increased risk behavior in persons with ADHD have focused on specific risk behaviors, such as substance use^5^,^6^,^7^, risky driving^8^, and risky sex behavior^10^. Only rarely, do studies on ADHD approach risk taking as a general tendency. Recently, we asked adolescents with and without ADHD to estimate the frequency with which they engaged in 16 different risk-taking behaviors. Total risk taking score (across all behaviors) was higher for adolescents with ADHD than for adolescents without ADHD^18^. The present study confirms the relation between ADHD and the tendency to engage in risk-taking behavior, both in general and in specific real-life domains, including health, recreational, financial and ethical. In contrast to other domains, the positive correlation between ADHD symptoms and risk taking in the social domain was not significant.

### ADHD symptoms and risk/benefit perceptions and attitudes

Benefit and risk perception were rarely examined in the ADHD literature. A main finding of the current study is that ADHD symptoms correlate with the perception of the benefits associated with the risky behaviors. Mediation analysis supported a model according to which ADHD symptoms lead to higher benefit perception, which in turn lead to greater engagement in risky behaviors.

Our findings are in accord with one study reporting that adolescents with clinical hyperactivity/impulsivity rates endorsed higher positive smoking expectancies^21^, but not with other studies reporting that individuals with ADHD had lower levels of positive alcohol expectancies compared with individuals without ADHD^22^, and that individuals with ADHD had lower marijuana expectancies regarding social enhancement and tension reduction^28^. Taking a broader point of view, enhanced perceptions of the benefits of risky behavior is in line with some characteristics the literature ascribes to ADHD decision making. For example, sensation seeking which could potentially enhance the assessment of the benefits^29^ was found to mediate ADHD-associated risk taking^30^. Similarly, delay aversion, which is known to affect ADHD-related behavior^31^, may enhance the perceived benefits of alternatives that do not involve waiting. Future studies should further investigate the conditions under which people with ADHD perceive risky behaviors particularly beneficial.

On the other hand, ADHD symptoms did not correlate with levels of risk perception in our study. This finding is not in accordance with other studies reporting that children with ADHD attributed less severe consequences to risky activities^19^, and disregarded the consequences of their risk taking^20^, that adolescents with clinical inattention rates endorsed less negative expectancies regarding cigarette smoking^21^, and that adults with ADHD had lower marijuana expectancies regarding cognitive and behavioral-impairment^28^. One important methodological difference between our study and the ones mentioned above concerns the measurement of perceptions. Whereas in other studies participants had to estimate the likelihood of specific consequences of substance use, in our study they had to indicate their general “gut level assessment” of the extent to which various behaviors are beneficial or risky. Possibly, these “gut level” perceptions of the benefit of outcomes, and not the likelihood of these outcomes, are more subjected to ADHD symptoms.

According to the BDT^11^, a distinction should be made between the perceptions of risk and benefit and the attitudes toward these perceptions. Using individual regression analyses, we computed the attitudes towards perceived-risk (risk aversion) and perceived-benefit (benefit seeking) for each participant. Surprisingly, level of ADHD symptoms had a negative correlation with benefit seeking. Together with the previous findings, it seems that stronger ADHD symptoms are associated with higher benefit perception (e.g., smoking cigarette is rated more highly) and lower benefit seeking (i.e., risk taking is less affected by benefit perception). This complex picture may correspond to the variety of findings regarding the complex behavioral and neural response to reward and cost contingencies^32^,^33^.

The lack of association between ADHD and increased risk seeking seems in accordance with another line of evidence. A popular procedure used for studying risk taking in ADHD involves laboratory-gambling tasks, where subjects are asked to choose between safe and risky alternatives. Groen, Gaastra, Lewis-Evans, and Tucha^34^ found in their review that “half of the studies in children/adolescents (50%), but only a minority of studies in adults (27%) reported greater risky performance in individuals with ADHD when compared to normal controls” (p. 13)^34^. Furthermore, those studies that have shown increased risk taking^35^ have often used tasks in which risk seeking and suboptimal decision-making were confounded (i.e., the riskier alternative was regularly less favorable in terms of its expected value), thus choosing the risky alternative could have reflected either risk seeking or poor decision-making. In a series of experiments conducted recently in our laboratory, we found no differences between ADHD and control groups in choosing between risky and safe alternatives that were equally favorable^18^. Such findings suggest that ADHD is not associated with increased risk seeking, and rather open up the possibility that ADHD involves some disruption in the perception of the choice outcomes, which may lead to non-optimal choice.

### Clinical implications

The investigation of the mechanisms underlying impaired decision making among people with ADHD has important clinical implications. Specifically, it informs prescriptive research with the goal of helping people with ADHD to optimize their decision-making and counter their engagement in dangerous activities. Our findings suggest that interventions aimed at reducing risk taking in adults should include measures of their ADHD symptoms as well as their perceptions of the benefits of engaging in risky behaviors. Interventions may be devised in light of the research, which would deal with external regulation and strategies that take into account the individuals’ preferences.

### Limitations

This study has several limitations: The convenience sampling resulted in limited age distribution and over-representation of individuals with higher education. However, education did not correlate with risk measures. Second, we measured hypothetical risk taking (and used scale ratings), rather than actual engagement in risky behavior. In addition, engagement in risky behaviors was assessed using self-report, which was not validated by collateral report. Yet, the present method focuses on participants’ motivations and perceptions regarding an unconstrained range of behaviors. Thus, we could elicit one’s attitude towards risky behaviors, such as riding a motorbike without a helmet regardless of whether one owns a motorbike or ever rides one in real life.

## Methods

### Participants

All experimental protocols were approved by the Shaare Zedek Medical Center Institutional Review Board for research on human subjects. The methods were carried out in accordance with the approved guidelines. Participants were recruited through advertisements in universities, colleges and work places. Gender composition was 54.1% females (*n* = 132) and 45.9% males. The sample’s mean age was 34.24 ± 11.78, and mean years of education was 14.50 ± 1.67. Twenty-three participants (9.4%) reported that they had been formally diagnosed with ADHD. Subjects did not receive monetary compensation for participation.

### Protocol and Measures

Meetings with participants took place in a quiet room. Written informed consent was obtained from all subjects, followed by completion of a demographic questionnaire, the Adult ADHD Self-Report Scale (ASRS) and the Domain Specific Risk-Taking (DOSPERT) scale.

Demographic questionnaire: Participants provided background information on age, gender, and history of diagnosis of ADHD.

The Hebrew version of the ASRS-V1.1^36^,^37^ is a dimensional measure of ADHD symptoms. It includes 18 items corresponding to the DSM diagnostic criteria of ADHD, each measured for its frequency on a Likert scale ranging from 1 (never) to 5 (very often). The questionnaire has high internal consistency (α = 0.88). As an ADHD screener, the scale’s sensitivity and specificity are 68.4% and 99.6%, respectively^38^.

Domain-Specific Risk-Taking (DOSPERT): Blais and Weber’s version of the DOSPERT (2006) scale assesses risk taking in five domains: health/safety, finance, ethical choices, social interaction, and recreation^24^. The DOSPERT measures risk taking, perceived benefits and risks of 30 activities, using seven-point Likert scales (risk-taking: 1 = extremely unlikely, 7 = extremely likely; benefit perception: 1 = no benefits, 7 = great benefits; risk perception: 1 = not at all risky, 7 = extremely risky). The three DOSPERT scales can each be broken apart into five subscales representing specific domains of risk-taking (i.e., ethical, financial, health and safety, recreational, social). Harrison, Young, Butow, Salkeld, and Solomon^39^ recommended the DOSPERT for its ability to assess risk taking in different everyday domains and to separate perceptual and attitudinal reasons for taking risks^39^. The scale has adequate internal-consistency and moderate test-retest reliability estimates, and it provided evidence for the factorial and convergent/discriminant validity of the scores with respect to constructs such as sensation seeking, dispositional risk taking, intolerance for ambiguity, and social desirability^11^. Construct validity was also assessed via correlations with the results of a risky gambling task as well as with tests of gender differences^11^. The DOSPERT scale was translated into Hebrew by our team employing scientific translation rules, including multiple translators and independent back-translations.

### Statistical Analysis

Scores for the ASRS were computed by summing responses to the 18 items together, as well as separately for inattention and for hyperactivity/impulsivity. Scores for risk-taking, benefit perception and risk perception were computed in two ways. First, by averaging the responses to all 30 behaviors and second, by averaging for each domain separately the responses to all six relevant behaviors. Using the BDT regression equation noted above for each participant, we calculated the two coefficients (*a* and *b*) which index the individual attitudes towards benefit and risk. Since each domain scale involved only six items, attitudes could not be reliably calculated with a separate regression for each individual and for each domain^40^. Observations that were more than 3.01 standard deviations away from the group mean were defined as outliers, according to Grubbs G outlier test^41^ for *N* > = 25 and alpha = 0.01. Outliers were replaced by the 3.0 SD values, according to the Winsorising method, as recommended by Tabachnick and Fidell^42^. This method of dealing with extreme case values preserves the increased value of the outlier sample, whilst ameliorating its disproportionate influence on the data. Preliminary analyses were conducted to examine the normative distribution of each variable using the Kolmogorov-Smirnov test, and to examine whether there were any statistically significant associations between demographic variables and our study variables.

Next, we examined correlations between the total ASRS score and the DOSPERT scores. Tests of significance were two-sided. Finally, direct and indirect effects of ADHD symptoms on risk-taking behavior were calculated using the multiple mediation approach and SPSS macro (PROCESS, Model 6) provided by Hayes^43^. Following procedures recommended by Preacher and Hayes^44^, a multiple mediation model involves (a) an analysis of the total indirect effect–the aggregate mediating effect of all the mediators being examined and (b) an analysis of the specific indirect effect–the mediating effect of a specific mediator^44^. The significance of the indirect effects was tested via bootstrap analysis, which is commonly performed in multiple mediator analyses given its advantage of greater statistical power without assuming multivariate normality in the sampling distribution, assuming only the sample is representative of the population^44^,^45^,^46^. Mediation is demonstrated via a statistically significant indirect effect (i.e., if the 95% bias-corrected confidence interval for the parameter estimate does not contain zero). All analyses were conducted using SPSS 21.0 including an SPSS macro designed for assessing multiple mediation models^44^. Additional analyses included separate correlation and mediation analyses of the relations between the specific ASRS scales, namely, inattention and hyperactivity/impulsivity, and the total DOSPERT score.

## Additional Information

**How to cite this article**: Shoham, R. *et al*. ADHD-associated risk taking is linked to exaggerated views of the benefits of positive outcomes. *Sci. Rep.* **6**, 34833; doi: 10.1038/srep34833 (2016).

## Figures and Tables

**Table 1:** Descriptive statistics of ASRS and DOSPERT scales.

	Median	25–75%
ASRS	43	37–50
inattention	22	18–27
hyperactivity	21	17–25
DOSPERT scales		
Risk-Taking	2.83	2.33–3.27
Benefit-Perception	2.67	2.20–3.16
Risk-Perception	4.33	3.87–4.80
Perceived-benefit Attitude	0.39	0.21–0.61
Perceived-risk Attitude	−0.34	−0.52–−0.22

*Note. N* = 244 (132 females, 112 males); ASRS, Adult ADHD Self Report Scale; DOSPERT, Domain-specific Risk-Taking.

**Table 2:** Correlations between the ASRS specific dimensions and the DOSPERT scales.

	ASRS total score	Inattention	Hyperactivity/Impulsivity	Risk-Taking	Benefit-Perception	Risk-Perception	Perceived-benefit Attitude	Perceived-risk Attitude
ASRS total score	—							
Inattention	0.883*	—						
Hyperactivity/Impulsivity	0.862*	0.563**	—					
Risk-Taking	0.268**	0.247**	0.219**	—				
Benefit-Perception	0.286**	0.289**	0.219**	0.717**	—			
Risk-Perception	−0.005	0.005	−0.002	−0.450**	−0.327**	—		
Perceived-benefit Attitude	−0.183*	−0.154*	−0.196**	−0.023	−0.164*	0.035	—	
Perceived-risk Attitude	−0.075	−0.085	−0.077	0.001	−0.073	−0.246**	0.533**	—

*Note. N* = 244 (132 females, 112 males); Correlation between Adult ADHD Self Report Scale (ASRS) total, inattention and hyperactivity/impulsivity scores and the Domain-specific Risk-Taking (DOSPERT) scales was conducted using Spearman’s rho test. ^*^p < 0.05, ^**^p < 0.01.

**Table 3:** Mediation models for general ASRS score and for separate ADHD dimensions.

ADHD dimension	Model R^2^	Indirect effect:				Direct effect
		Benefit Perception	Risk Perception	Perceived-benefit Attitude	Perceived-risk Attitude	
Total	57.3	0.184* 95% CI [0.116, 0.224]	0.004 95% CI [−0.028, 0.041]	−0.037 95% CI [−0.085, −0.008]	0.006 95% CI [−0.006, 0.034]	0.113* 95% CI [0.020, 0.205]
Inattention	56.8	0.184* 95% CI [0.114, 0.273]	0.004 95% CI [−0.029, 0.040]	−0.029 95% CI [−0.077, −0.004	0.004 95% CI [−0.004, 0.003]	0.084 95% CI [−0.009, 0.176]
Hyperactivity/Impulsivity	55.3	0.151* 95% CI [0.079, 0.230]	0.006 95% CI [−0.028, 0.043]	−0.036 95% CI [−0.088, −0.006]	0.006 95% CI [−0.006, 0.038]	0.106* 95% CI [0.016, 0.197]

Final mediation path analysis predicting risky behavior separately in each domain. Values reflect standardized regression coefficients of indirect effects and of direct effect (after considering other mediators) of total and subscale scores of ASRS on risky behavior. The covariates of age and gender are not shown in for visual clarity. *N* = 244 (132 females, 112 males).

**Table 4:** Mediation models for separate risk taking domains.

DOSPERT domain	Model R^2^	Indirect effect:		Direct effect
		Benefit Perception	Risk Perception	
Total score	55.6	0.184* 95% CI [0.050, 0.139]	0.004 95% CI [−0.028, 0.041]	0.006 95% CI [−0.001, 0.012]
Health/Safety	48.6	0.092* 95% CI [0.043, 0.208]	0.034 95% CI [−0.023, 0.051]	0.178* 95% CI [0.080, 0.275]
Recreational	62.3	0.117* 95% CI [0.043, 0.208]	0.010 95% CI [−0.023, 0.051]	0.008 95% CI [−0.074, 0.091]
Ethical	29.3	0.110* 95% CI [0.042, 0.172]	0.009 95% CI [−0.010, 0.049]	0.133 95% CI [−0.009, 0.206]
Financial	35.2	0.099* 95% CI [0.052, 0.212]	0.010 95% CI [−0.023, 0.051]	0.098* 95% CI [0.016, 0.251]
Social	50.2	0.046 95% CI [−0.024, 0.120]	−0.049* 95% CI [−0.098, −0.016]	0.069 95% CI [−0.025, –0.164]

Final mediation path analysis predicting risky behavior separately in each domain. Values reflect unstandardized regression coefficients of indirect effects and of direct effect (after considering other mediators) of ADHD on risky behavior. The covariates of age and gender are not shown in for visual clarity. *N* = 244 (132 females, 112 males).
